# Improving ECG Competence in Medical Trainees in a UK District General Hospital

**DOI:** 10.14740/cr333e

**Published:** 2014-05-15

**Authors:** Christopher McAloon, Helen Leach, Simrat Gill, Arun Aluwalia, Jasper Trevelyan

**Affiliations:** a Worcestershire Acute Hospitals NHS Trust, Charles Hastings Way, Worcester, WR5 1DD, UK; b Birmingham Heartlands Hospital NHS Trust, Bordesley Green, Birmingham, B9 5SS, UK

**Keywords:** ECG, Interpretation, Competence, Confidence, Training

## Abstract

**Background:**

Competency in electrocardiogram (ECG) interpretation is central to undergraduate and postgraduate clinical training. Studies have demonstrated ECGs are interpreted sub-optimally. Our study compares the effectiveness of two learning strategies to improve competence and confidence.

**Method:**

A 1-month prospective randomized study compared the strategies in two cohorts: undergraduate third year medical students and postgraduate foundation year one (FY1) doctors. Both had blinded randomization to one of these learning strategies: focused teaching program (FTP) and self-directed learning (SDL). All volunteers completed a confidence questionnaire before and after allocation learning strategy and an ECG recognition multiple choice question (MCQ) paper at the end of the learning period.

**Results:**

The FTP group of undergraduates demonstrated a significant difference in successfully interpreting “ventricular tachycardia” (P = 0.046) and “narrow complex tachycardia” (P = 0.009) than the SDL group. Participant confidence increased in both learning strategies. FTP confidence demonstrated a greater improvement than SDL for both cohorts.

**Conclusion:**

A dedicated teaching program can improve trainee confidence and competence in ECG interpretation. A larger benefit is observed in undergraduates and those undertaking a FTP.

## Introduction

**Figure 1:**
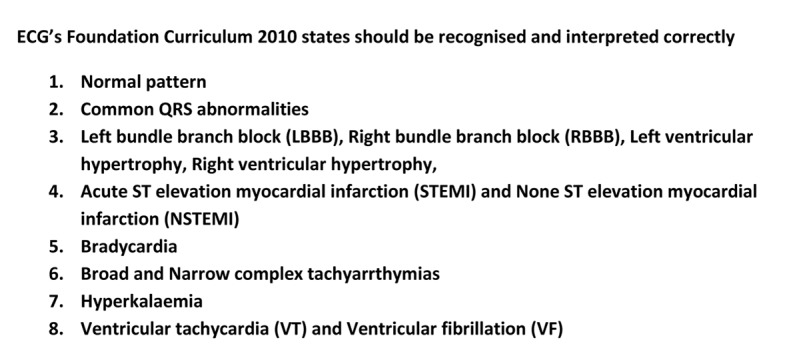
Foundation Curriculum 2010.

The electrocardiogram (ECG) is the most commonly used diagnostic test in modern medicine [1]. ECG interpretation forms a core investigative skill of the physician [2, 3]. It is essential for undergraduate and postgraduate trainees to become competent in this skill [3]. British postgraduate training lists ECG interpretation as a core skill and one where competence needs to be achieved within the first year of practice. The Foundation Curriculum 2010 (FC 2010) used by all British foundation doctors stipulates that they must be able to “recognize and interpret ECGs” [4]. There are specific abnormalities on 12-lead ECGs and rhythm strips that should be interpretable to be deemed competent (Fig. 1).

Despite the emphasis put on ECG interpretation in the FC 2010, many do not achieve the desired standard [4]. Multiple studies demonstrate that many foundation year one (FY1) doctors, senior house officers and attending physicians interpreted ECGs incorrectly [5, 6]. In 2000, a British study of 46 graduated final year medical students were tested on recognition of several, common ECG findings including those in the FC 2010 [4, 7]; 9% rated themselves as “comfortable” with ECG interpretation [7]. Complete heart block, atrial fibrillation and inferior myocardial infarction were only identified correctly in 67%, 54% and 61% respectively. This study did not include a clinical history with the ECG and this can improve interpretation by 4-12% [8]. A retrospective analysis of 1,000 emergency department cases and ECGs found that 38 patients had been discharged with “abnormalities that could potentially alter case management” [9].

Undergraduate and postgraduate training does not consistently deliver satisfactory competency in ECG interpretation. Traditional learning strategies alone are not achieving curriculum objectives. Mahler et al considered whether ECG interpretation was better taught through self-directed learning (SDL), lectures or workshops for American medical students [10]. Two hundred and twenty-three students were randomized to one of these three learning strategies [10]. All formats demonstrated some improvement in educational scores. However lectures and workshops improved scores significantly more than SDL format alone [10].

Current learning strategies may not be not succeeding in producing undergraduate or postgraduate medical trainees competent in ECG interpretation. There may be an overreliance on SDL, and alone this may not always allow medical trainees to achieve competence. Alternative learning strategies should be evaluated and analyzed.

## Aims

1) To assess the effect of two different learning strategies (focused teaching program (FTP) and SDL) on confidence in ECG interpretation for undergraduate and postgraduate medical trainees.

2) To assess the effect of two different learning strategies (FTP and SDL) on competence in ECG interpretation for undergraduate and postgraduate medical trainees.

## Method

A prospective randomized study was carried out over a 1-month period comparing two learning strategies in two cohorts: undergraduate third year medical students (first clinical rotations) and postgraduate FY1 doctors. Both cohorts had blinded randomization to one of these learning strategies: FTP and SDL. All participants volunteered prior to randomization. The study took place between October and November 2012. Randomization was performed using sealed envelopes. All groups were instructed to continue with their normal educational programs during the period of learning strategy implementation. The FTP was designed and delivered by one author (CM), who was blinded to randomization.

Prior to undergoing either learning strategy, all participants were invited to complete a questionnaire on confidence and competence. The questionnaire was online (www.surveymonkey.com) to increase response rate. Following completion of the assigned learning strategy, participants completed a repeat confidence and competence questionnaire. Additionally under examination conditions, the volunteers were asked to complete a multiple choice questionnaire (MCQ) on ECG recognition for objective assessment of competence. The MCQ was set by two independent consultant cardiologists aware of the FC 2010. The MCQ difficulty was altered to an appropriate standard for each cohort.

The FTP groups (one per cohort) undertook a series of seminars focusing on becoming competent on ECG interpretation to the FC 2010 standard [4]. The FY1 group had three 1-h sessions, the third year medical students had four 1-h sessions. Topic areas covered were “ECG basics”, “ischemia”, “arrhythmias” and “special circumstances”. There was a workbook used in support of the arrhythmia seminar. SDL groups were provided with objectives but had no formal teaching. All participants were told to continue with their educational routine and attend the normal educational programme.

### Ethical approval

Involvement in the study was on a voluntary basis. Permission was given by the Foundation Year One Supervisor and the Undergraduate Dean for the local district general hospital.

### Statistical analysis

Statistical analysis was performed using STATA 11.2 (StataCorp, Texas). The objective assessment was analyzed using a two-tailed *t*-test with a confidence interval set at 95%.

## Results

There were 26 FY1 doctors and 36 third year medical students based at Worcester Royal Hospital during the study period. All were invited to participate in the study. Twenty-one FY1s and 25 third year medical students volunteered, representing an 80.1% and 69.4% response rate respectively. Randomization allocated volunteers to either intervention group: FY1 (FTP 11, SDL 10 ) and third year medical students (FTP 13, SDL 12).

### Demographics

Table 1 demonstrates the demographics of each group within each cohort.

**Table 1:** Demographics of the Volunteers

	Third year medical students (25)		FY1 (21)	
	FTP (13)	SDL (12)	FTP (11)	SDL (10)
Gender % (no. of male)	15.4% (2)	38.5% (5)	65.5% (6)	45.5% (5)
Age range (mean)	20 - 31 (22)	20 - 30 (21)	23 - 33 ( 25)	23 - 28 (26)

FTP: focused teaching program; SDL: self-directed learning; FY1: foundation year one.

### Previous ECG interpretation learning

Thirty-six percent (n = 9) of the third year medical students had undergone formal ECG teaching. Thirty-two percent (n = 8) listed the educational format as “lectures”.

All FY1s had previous formal training in ECG interpretation. Seventy-three point six percent (n = 14) of FY1s had previously undertaken SDL. The commonest educational format for 84% (n = 16) was “small group seminars” followed by “lecture” for 47% [9]. None of the FY1 cohort had “e-learning” experience.

### Attendance in FTP

There were four specific seminars for the third year medical student FTP group; 76.9% (n = 10) attended all four sessions, with 25.4% (n = 3) attending three sessions. Attendance was impacted by other teaching and days not on placement.

The FY1s had three specific sessions allocated to their FTP group. There was a reduced attendance with 40% (n = 4) attending three sessions, 40% (n = 4) attending two and 20% (n = 2) attending one session. The FY1s had a lower attendance due to annual leave, on-call (day or night) or had other commitments.

### SDL study time

Fifty percent (n = 6) of the third year medical students SDL group spent 1 - 2 h on SDL. One student did not do any SDL during the observation period.

The FY1 SDL group all spent at least 1 h learning about ECG interpretation. The maximum time spent was 1 - 2 h which was performed by 36.4% (n = 4).

### Confidence in ECG interpretation

**Figure 2:**
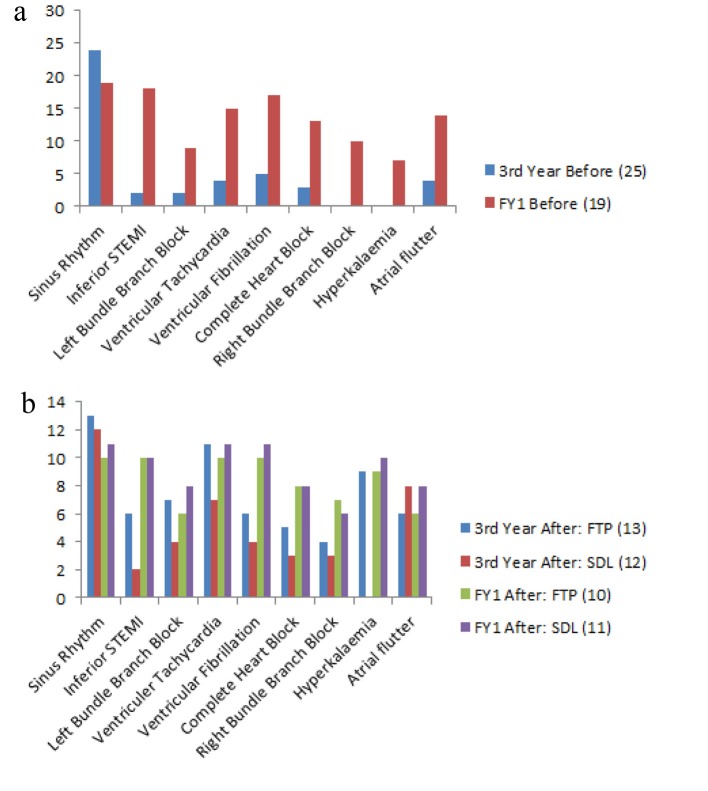
(a) Individual ratings whether they are confident in being able to interpret specfic ECGs before undergoing learning strategy. (b) Individual ratings whether they are confident in being able to interpret specfic ECG after undergoing learning strategy.*Total initial FY1 response rate reduced as two members cohort did not respond. FTP: focused teaching program; SDL: self-directed learning; FY1: foundation year one.

The same confidence questionnaire was used before and after the interventions. Table 2 demonstrates participant’s perception of their own confidence in ECG interpretation. Comparison is made within groups between each cohort undergoing different learning strategies. Figure 2 demonstrates participant’s perception of their own confidence to interpret specific ECGs (specific 12-lead ECG and rhythm strips) before and after the undertaken learning strategy. The perception of cohort confidence increased for all third year medical students, with a greater improvement in the FDL group for all ECGs. The FY1s had had a small increase in their confidence in both groups with no difference between learning strategy.

**Table 2:** Confidence Ratings Before and After Learning Strategy

Confidence rating	Third year students			FY1 doctors		
	Before (25)	After FTP (13)	After SDL (12)	Before (21)	After FTP (10)	After SDL (11)
Very poor	60% (15)	0% (0)	16.7% (2)	0% (0)	0	0
Poor	36% (9)	38.5% (5)	75% (9)	19.1% (4)	10% (1)	27.3% (3)
Satisfactory	4% (1)	46.2% (6)	8.3 % (1)	66.6% (14)	60% (6)	72.7% (8)
Good	0 (0)	7.7% (1)	0% (0)	4.8% (1)	30% (3)	0% (0)
No response	0 (0)	7.7% (1)	0% (0)	9.5% (2)	0% (0)	0% (0)

FTP: focused teaching program; SDL: self-directed learning; FY1: foundation year one.

Participants were asked to state how often they needed to ask for clarification regarding their interpretation of an ECG, before and after learning strategy. Table 3 outlines the frequency needed for clarification before and after learning strategy.

**Table 3:** Frequency of Clarification About ECG

Frequency of asking for help	Third year students			FY1 doctors		
	Before (25)	After FTP (13)	After SDL (12)	Before (21)	After FTP (10)	After SDL (11)
Never	0% (0)	0% (0)	8.3% (1)	0% (0)	0% (0)	0
Occasionally	4% (1)	15.4% (2)	8.3% (1)	4.7% (1)	80% (8)	63.6% (6)
Often	8% (2)	46.2% (6)	25% (3)	33.3% (7)	20% (2)	36.4% (3)
Always	88% (22)	38.5% (5)	58.3% (7)	52.3% (11)	0% (0)	0
No response	0% (0)	0% (0)	0% (0)	9.5% (2)	0% (0)	0% (0)

FTP: focused teaching program; SDL: self-directed learning; FY1: foundation year one.

### Objective assessment of ECG interpretation

Participants underwent an MCQ assessment of their ECG interpretation competence. The third year medical students and FY1s were asked about the same ECG for each question, but questions were worded differently between each cohort based on the standard expected at that level. Table 4 represents number of correct answers per question and statistical analysis between cohort’s responses. For individual ECG interpretation, there were no significant differences between each FY1 group. The third medical students in the FTP group interpreted “ventricular tachycardia” and “regular narrow complex tachycardia” significantly better than their SDL counterparts.

**Table 4:** MCQ Successful Response Rate

ECGs interpreted	Third year medical students (n = 25)			FY1 (n = 21)		
	FTP: correct responses (n = 13)	SDL: correct responses (n = 12)	P value	FTP: correct responses (n = 10)	SDL: correct responses (n = 11)	P value
Ventricular tachycardia (Q1)	92.2% (12)	58.3% (7)	0.0469	100% (10)	90.9% (10)	0.3286
Atrial fibrillation (Q2)	61.5% (8)	58.3% (7)	0.8702	100% (10)	100% (11)	n/a
Inferior STEMI (Q3)	92.2% (12)	91.6% (11)	0.9529	90% (9)	100% (11)	0.2825
Regular narrow complex tachycardia (Q4)	76.9% (10)	25% (3)	0.0094	100% (10)	90.9% (10)	0.3286
Complete heart block (Q5)	23.1% (3)	33.3% (4)	0.5683	80% (8)	81.8% (9)	0.9156
Atrial flutter 4:1 block (Q6)	92.2% (12)	91.6% (11)	0.9529	80% (8)	81.8% (9)	0.9156
Hyperkalaemia (Q7)	76.9% (10)	83.3% (10)	0.6889	100% (10)	90.9% (10)	0.3286
Right bundle branch block (Q8)	46.2% (6)	41.7% (5)	0.8213	100% (10)	90.9% (10)	0.3286
Left bundle branch block (Q9)	23.1% (3)	41.7% (5)	0.3195	50% (5)	45.5% (5)	0.835
First degree heart block (Q10)	23.1% (3)	33.3% (4)	0.5683	80% (8)	90.9% (10)	0.4755
Wenkebach - second degree heart block (Q11)	61.5% (8)	33.3% (4)	0.1585	60% (6)	54.5% (6)	0.8008

FTP: focused teaching program; SDL: self-directed learning; FY1: foundation year one.

## Discussion

ECG interpretation is a core clinical skill required as a doctor. Junior doctor’s confidence and competence has been demonstrated to be unsatisfactory, with potential poor diagnostic and management skills as a result [5, 6, 9]. Undergraduates do not always receive teaching on ECG interpretation and this can impact confidence [7]. The effectiveness of formal teaching is better than SDL alone [10]. This problem is recognized in the UK. The FC 2010 emphasizes the importance of obtaining this core skill at an early stage in postgraduate training [4].

In a UK district general hospital, we have demonstrated a low level of confidence in ECG interpretation at the undergraduate and postgraduate level. The low level of confidence in the undergraduates at the start of their first clinical rotation could be expected as there may have been limited prior teaching on ECG interpretation. The postgraduate participant’s low level of confidence in ECG interpretation is a greater concern. Postgraduates were more confident at recognizing life-threatening ECG rhythms (ventricular tachycardia and fibrillation) than other common ECG rhythms (complete heart block, atrial flutter). A possible explanation for this could be the recent attendance at the advanced life support course. FY1s achieved a high level of correct interpretation on the MCQ reflecting the improved self-rated confidence for life-threatening rhythms. Other rhythms outlined in the FC 2010 (right and left bundle branch block) were not interpreted correctly by many FY1 participants.

Overall confidence improved for all participants, with a greater improvement in the undergraduate group. The FTP cohort demonstrated a small improvement in confidence in the postgraduate group and a larger improvement in the undergraduate group. The undergraduate FTP cohort demonstrated a greater improvement in confidence for recognition of all ECGs and rhythms (except atrial flutter) than the SDL cohort. Postgraduate cohorts demonstrate no difference in confidence for specific ECG recognition.

The MCQ performance for the undergraduates demonstrated a difference between both cohorts in their level of successful ECG interpretation. The FTP cohort achieved better marks for several ECGs including significantly better for ventricular tachycardia and regular narrow complex tachycardia. The workbook covers these ECGs in detail and may offer an explanation to the reason why the FTP group obtained higher scores. The postgraduates achieved similar success rates of correct interpretation on the MCQ irrelevant of which learning strategy they were assigned to. Both cohorts achieved a high correct interpretation rate for life-threatening rhythms. However, both cohorts had a lower interpretation rate for left bundle branch block and second degree heart block.

The study is limited due to low numbers of participants involved. Additionally attendance rates at all sessions were variable due to other factors. A further study on a larger scale would minimize these factors.

The study demonstrates baseline levels of confidence and competence in ECG interpretation stands in a modern cohort of undergraduates at the start of their clinical experience and postgraduates. The level of perceived confidence amongst postgraduate doctors (FY1) may be of concern. The objective assessment of competence is greater than perceived confidence for most specific ECGs. Undergraduates demonstrated the greatest improvement in confidence and competence having undergone an FTP. A focused teaching strategy benefits undergraduates with less experience than postgraduates. SDL alone for undergraduates does not maximize the potential benefits in achieving learning objectives. Postgraduates seem to benefit less from FTP, but there is a confidence improvement that should not be underestimated. A cost neutral FTP strategy offers the potential for doctors to achieve the acquired standard in ECG interpretation in both undergraduate and postgraduate training. 
